# Exosomes from umbilical cord-derived mesenchymal stem cells combined with gelatin methacryloyl inhibit vein graft restenosis by enhancing endothelial functions

**DOI:** 10.1186/s12951-023-02145-1

**Published:** 2023-10-18

**Authors:** Yuhang Deng, Yiming Li, Zhuyang Chu, Chun Dai, Jianjun Ge

**Affiliations:** Department of Cardiac Surgery, The First Affiliated Hospital of USTC, Division of Life Sciences and Medicine, University of Science and Technology of China, Hefei, 230001 Anhui China

**Keywords:** Intimal hyperplasia, Restenosis, Exosomes, Mesenchymal stem cells, Photosensitive hydrogel

## Abstract

**Background:**

The prevalence of coronary artery disease is increasing. As a common treatment method, coronary artery bypass transplantation surgery can improve heart problems while also causing corresponding complications. Venous graft restenosis is one of the most critical and intractable complications. Stem cell-derived exosomes could have therapeutic promise and value. However, as exosomes alone are prone to inactivation and easy removal, this therapeutic method has not been widely used in clinical practice. Methacrylated gelatin (GelMA) is a polymer with a loose porous structure that maintains the biological activity of the exosome and can control its slow release in vivo. In this study, we combined human umbilical cord mesenchymal stem cell-derived exosomes (hUCMSC-Exos) and GelMA to explore their effects and underlying mechanisms in inhibiting venous graft restenosis.

**Results:**

Human umbilical cord mesenchymal stem cells (hUCMSCs) were appraised using flow cytometry. hUCMSC-Exos were evaluated via transmission electron microscopy and western blotting. hUCMSC-Exos embedded in a photosensitive GelMA hydrogel (GelMA-Exos) were applied topically around venous grafts in a rat model of cervical arteriovenous transplantation, and their effects on graft reendothelialization and restenosis were evaluated through ultrasonic, histological, and immunofluorescence examinations. Additionally, we analyzed the material properties, cellular reactions, and biocompatibility of the hydrogels. We further demonstrated that the topical application of GelMA-Exos could accelerate reendothelialization after autologous vein transplantation and reduce restenosis in the rat model. Notably, GelMA-Exos caused neither damage to major organs in mice nor excessive immune rejection. The uptake of GelMA-Exos by endothelial cells stimulated cell proliferation and migration in vitro. A bioinformatic analysis of existing databases revealed that various cell proliferation and apoptosis pathways, including the mammalian target of rapamycin (mTOR)–phosphoinositide 3-kinase (PI3K)–AKT signaling pathways, might participate in the underlying regulatory mechanism.

**Conclusions:**

Compared with the tail vein injection of hUCMSC-Exos, the local application of a mixture of hUCMSC-Exos and GelMA was more effective in promoting endothelial repair of the vein graft and inhibiting restenosis. Therefore, the proposed biomaterial-based therapeutic approach is a promising treatment for venous graft restenosis.

## Background

The incidence of cardiovascular disease has been increasing in recent years. Coronary artery disease has become an important “killer” that threatens the health of middle-aged and elderly people. In clinical practice, autogenous vein coronary artery bypass grafting (CABG) is often used to treat atherosclerotic heart disease (1). However, venous graft restenosis is a common postoperative complication that can have serious consequences. Studies have shown that approximately 15–30% of patients develop symptoms of venous obstruction within one year of hospital discharge (2). Therapeutic approaches to resolve venous graft restenosis can effectively prevent the complications of a second operation in patients with coronary artery disease.

Mesenchymal stem cells (MSCs) are multi-lineage cells, which can self-renew and differentiate into various tissue cells (such as adipocytes and chondrocytes) and are active “star” cells in the field of regenerative medicine (3). Moreover, existing studies have shown that MSCs have paracrine functions and play a key role in MSC-based therapies (4). Two main paracrine pathways are associated with MSCs: the secretion of cytokines and the secretion of extracellular vesicles (EVs) (5). EVs contain various signaling factors, for example ribonucleic acid (RNA), microRNA (miRNA), and proteins, which can play regulatory roles through humoral transport throughout the body (6). Moreover, cell-free treatment with EVs offers better stability and safety than direct stem cell transplantation (7). Recently, MSC-derived exosomes have been found to restrain restenosis in venous grafts (6). However, there are difficulties in the utilization of MSC-derived exosomes for the treatment of venous graft restenosis because exosomes are easily deactivated by environmental factors, such as temperature, and are easy to be removed in vivo. Therefore, extending the retention time of exosomes around venous grafts by combining them with biomaterials, without disturbing their biological function, has become a research hotspot in biomedical engineering.

Hydrogels are porous, loose structures formed from naturally derived or synthetic polymers, which usually have excellent swelling properties and certain mechanical properties, thus playing an important role in medical fields, such as tissue repair and remodeling (8). Therefore, hydrogels are a promising substitute for extracellular matrices as a good scaffold or carrier for maintaining the functions of stem cells and their derivatives in vivo (9). Compared to traditional oral or subcutaneous routes, hydrogels carrying therapeutic factors can evade metabolic activity in the liver and other tissues, resulting in better therapeutic effects (10). Furthermore, inclusion of exosomes in the hydrogel resulted in a substantial reduction in the exosome degradation rate in vitro (11). In addition, therapeutic factors in exosomes can be directly delivered locally when exosomes are bound to hydrogels and applied near the target tissue (12). Shi et al. combined human gingival MSC-derived exosomes with hydrogels and applied them around the wound tissue in rats with diabetes to improve wound healing (13). Moreover, a photoinduced crosslinking hydrogel was found to be effective in maintaining the activity of exosomes, and application of this combination to the disease model of bone and joint defects yielded good results (14). Although exosome-supported hydrogels have been used in various disease models, such as those of tissue damage and joint repair, only few studies have combined cord blood MSCs with hydrogels to treat restenosis in venous grafts.

Compared with MSCs from other sources (such as bone marrow), human umbilical cord mesenchymal stem cells (hUCMSCs) have the following advantages: noninvasive access, rapid self-renewal and proliferation, and low immunogenicity (15). Therefore, we aimed to develop a biocompatible hydrogel capable of carrying hUCMSC exosomes (hUCMSC-Exos) to inhibit intimal hyperplasia in venous grafts. Methacrylated gelatin (GelMA) is a hydrogel that can be polymerized via a free-radical reaction after irradiation with ultraviolet light in the presence of a photoinitiator (16). Compared to other polymerization methods, photopolymerization has numerous advantages, such as ease of use, plasticity according to the mold, and certain mechanical properties (17). Moreover, GelMA has a porous structure that can effectively preserve drug factors and prolong the half-life of drug release (18). Given these properties, we hypothesized that GelMA would retain and consistently release hUCMSC-Exos directly around the intravenous graft, thereby promoting endothelial cell functions and inhibiting intimal hyperplasia (19). To this end, we incorporated hUCMSC-Exos into GelMA, molded and wrapped it around the venous graft, and removed the blood vessels 28 d later. We then investigated whether the combination of exosomes and hydrogels could inhibit endovascular hyperplasia and improve hemodynamics in a rat model of venous transplantation.

## Results

### Characterization of hUCMSCs and hUCMSC-Exos

**Fig. 1:**
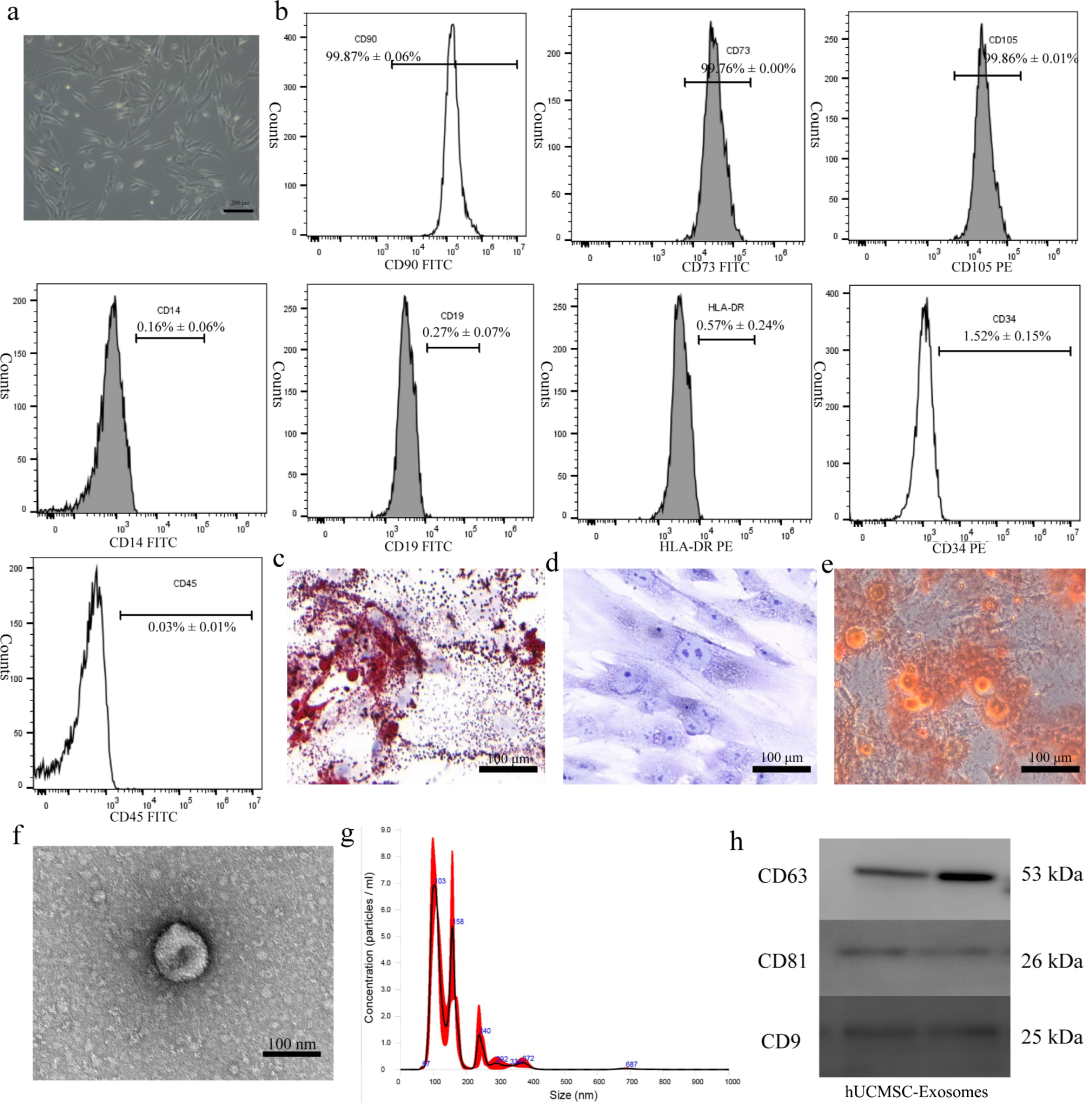
Morphology and characteristics of human umbilical cord mesenchymal stem cells (hUCMSCs) and hUCMSC exosomes. **a** Appearance of MSC fibroblast sample, as shown based on an optical microscope image (×40). **b** The surface markers of hUCMSCs were analyzed via flow cytometry. hUCMSCs tested positive for CD73, CD105, and CD90, but were negative for CD34, CD14, CD19, CD45, and HLA-DR. **c** MSCs had the ability to differentiate into adipocytes (×40). **d** MSCs also had the ability to differentiate into chondrocytes (×40). **e** MSCs had the ability to differentiate into osteoblasts (×40). **f** Exosome morphology observed via transmission electron microscopy. **g** The particle size distribution of exosomes measured using NANOSIGHT software. **h** Western blot analyses of the exosome surface markers (CD9, CD81, and CD63)

The typical morphology of fibroblasts was observed in vitro using optical microscopy (Fig. 1a). Flow cytometry analysis revealed that the cells were positive for CD73, CD90, and CD105 but constantly negative for CD34, CD45, CD14, CD19 and HLA-DR, which is consistent with a previous report of hUCMSC characteristics (Fig. 1b) (20). After two to three weeks of differentiation, the adipogenic and chondrogenic differentiation potential of hUCMSCs was confirmed by Alcian blue and Oil Red O staining (Fig. 1c, d). Also, hUCMSCs had the ability to differentiate into osteoblasts (Fig. 1e). The results showed that the cells used were hUCMSCs, which had the ability to differentiate. We then examined the morphology of the harvested exosomes using transmission electron microscopy (TEM). Under TEM, hUCMSC-Exos were observed to be elliptical in shape and variable in size (Fig. 1f). By testing the particle sizes of exosomes, we determined that their diameter was approximately 150 ± 77 nm (Fig. 1g). Furthermore, western blot analysis was performed to detect the expression of cluster of differentiation CD9, CD63, and CD81 (Fig. 1h).

### Characterization of GelMA and GelMA-Exos

**Fig. 2:**
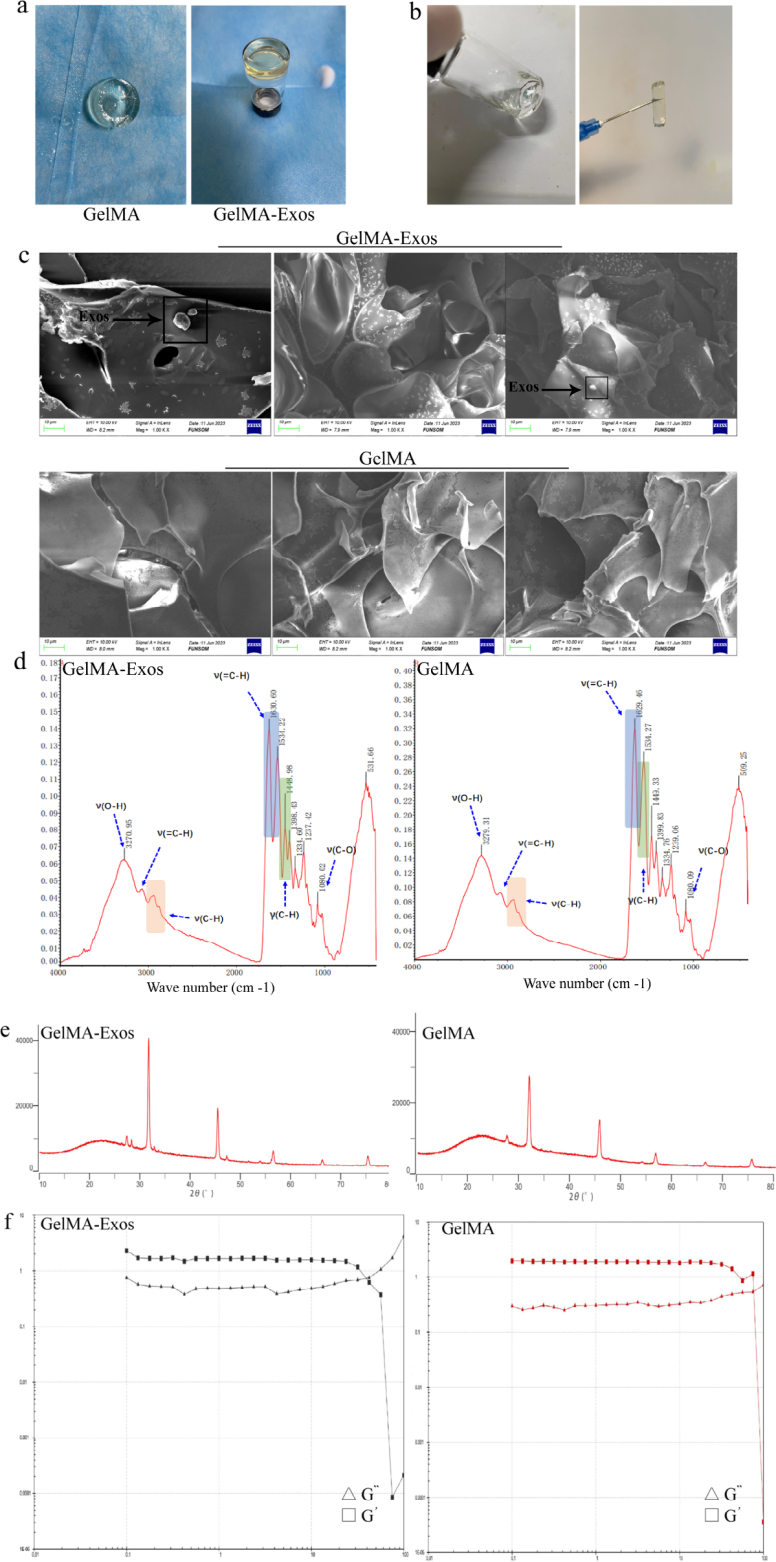
Physicochemical characterization of methacrylated gelatin-exosomes (GelMA-Exos) and GelMA. **a** The GelMA hydrogel was colorless and transparent. **b** GelMA hydrogels could be applied via a syringe. **c** GelMA and GelMA-Exos observed via scanning electron microscope. **d** A Fourier transform infrared spectrometer (FTIR) was used for GelMA hydrogels and GelMA-Exos separately. **e** X-ray diffraction (XRD) tests were performed on GelMA hydrogels and GelMA-Exos separately. **f** The storage modulus and loss modulus of GelMA and GelMA-Exos measured using a rheometer

Both GelMA and GelMA-Exos were colorless and existed in a non-liquid form (Fig. 2a). We evaluated the injectivity and viscosity of the hydrogel using a syringe (Fig. 2b). The microstructures of GelMA and GelMA-Exos were observed using scanning electron microscopy (SEM) (Fig. 2c). We found that GelMA has a relatively typical loose, porous structure. In the internal structure of GelMA-Exos, exosomes exhibit a scattered distribution. Fourier-transform infrared spectrophotometry was used to test the GelMA pure gel and GelMA-Exos after the introduction of exosomes. The Fourier spectra of the two groups overlapped considerably, and their peaks were not significantly different (Fig. 2d). By analyzing the infrared absorption characteristic peaks of GelMA-Exos and GelMA, we found that there was no significant difference in the types and quantities of internal functional groups between them. This suggests that exosomes do not chemically react with the gels. We also characterized the pure GelMA gel and GelMA-Exos using X-ray diffraction and found that the peaks of the two sets of curves almost coincided, indicating that exosomes also had no effect on the internal hydrogel structure (Fig. 2e). The rheological properties of GelMA and GelMA-Exos were examined. We found that the storage modulus (G’) of GelMA-Exos was slightly higher than the loss modulus (G”), like in the pure GelMA group, indicating that GelMA remains stable before and after the addition of exosomes. The rheological data show that the loss modulus exceeds the energy storage modulus only when the angular frequency is greater than 75 rad/s (Fig. 2f). Rats and normal people can only achieve a state of motion at a limited angular frequency; thus, the movement of water gel in rats or a normal person remains stable.

### Degradation of GelMA and GelMA-Exos in vivo and in vitro

**Fig. 3:**
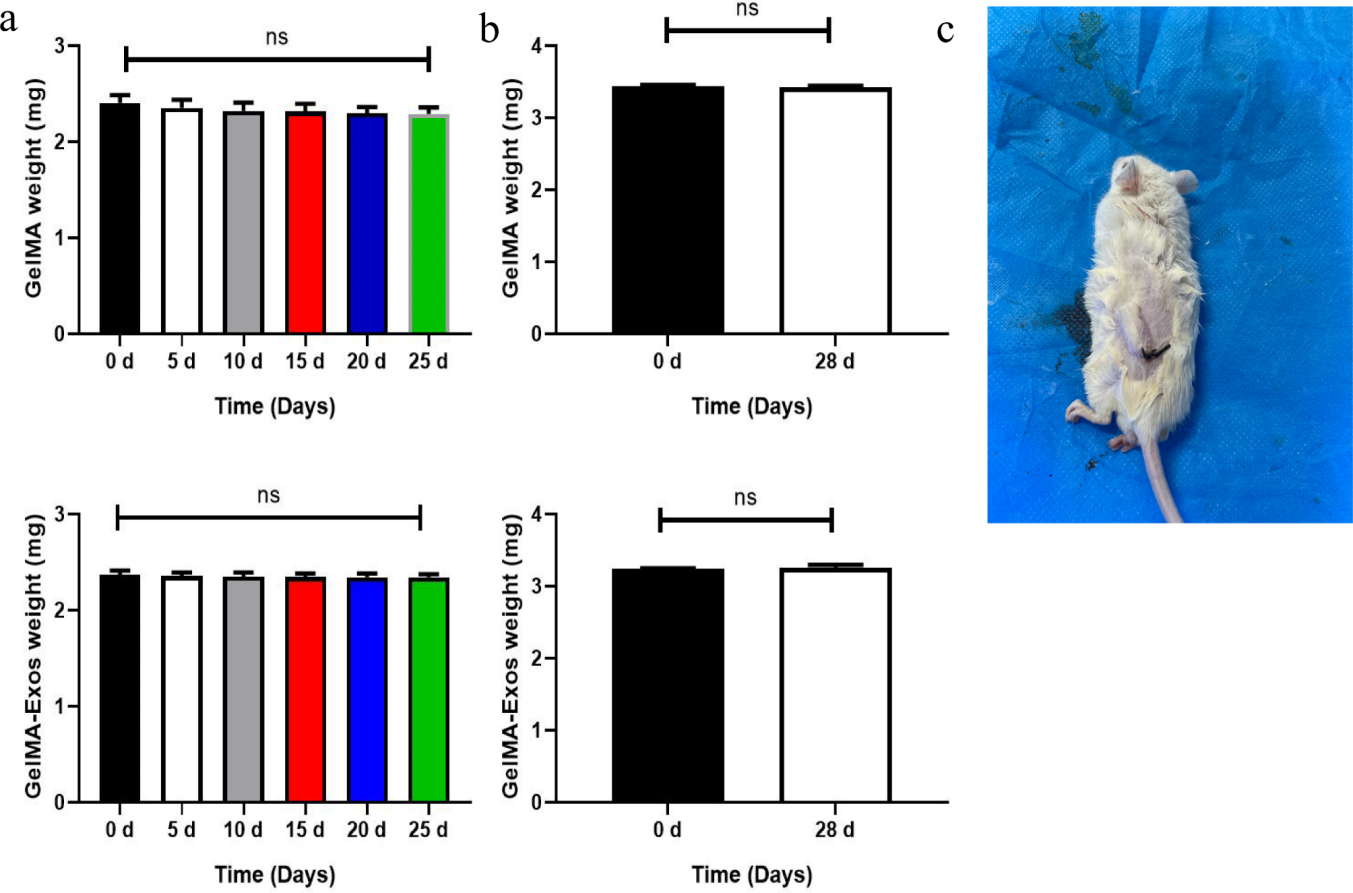
Degradation properties of methacrylated gelatin-exosomes (GelMA-Exos) and GelMA in vivo and in vitro. **a** Every five days, the weights of GelMA-Exos were measured. **b** GelMA-Exos were implanted subcutaneously into mice and taken out and weighed after 28 d. **c** The hydrogel was implanted under the skin of the backs of mice. Data are presented as the mean ± standard deviation (SD). Non-significant (ns): compared with 0 d, *P* > 0.05

To analyze the degradation of the hydrogels in vitro, we steeped them in phosphate buffered saline (PBS), harvested them every five days, and weighed them. GelMA and GelMA-Exos had initial weights of 2.407 ± 0.08 mg and 2.370 ± 0.04 mg, respectively. We plotted the degradation curves of GelMA and GelMA-Exos in vitro and found no significant differences in the weights between GelMA and GelMA loaded with exosomes (Fig. 3a). GelMA and GelMA-Exos with initial weights of 3.446 ± 0.01 mg and 3.248 ± 0.00 mg, respectively, were implanted under the skin of the mice’s backs. Subsequently, GelMA was collected and weighed after 28 d of implantation in rats, and it was found that its weight decreased slightly, with no significant difference being observed before and after GelMA was implanted in mice (Fig. 3b and c). Similar results were found for GelMA-Exos.

### Biocompatibility of GelMA in the body

**Fig. 4:**
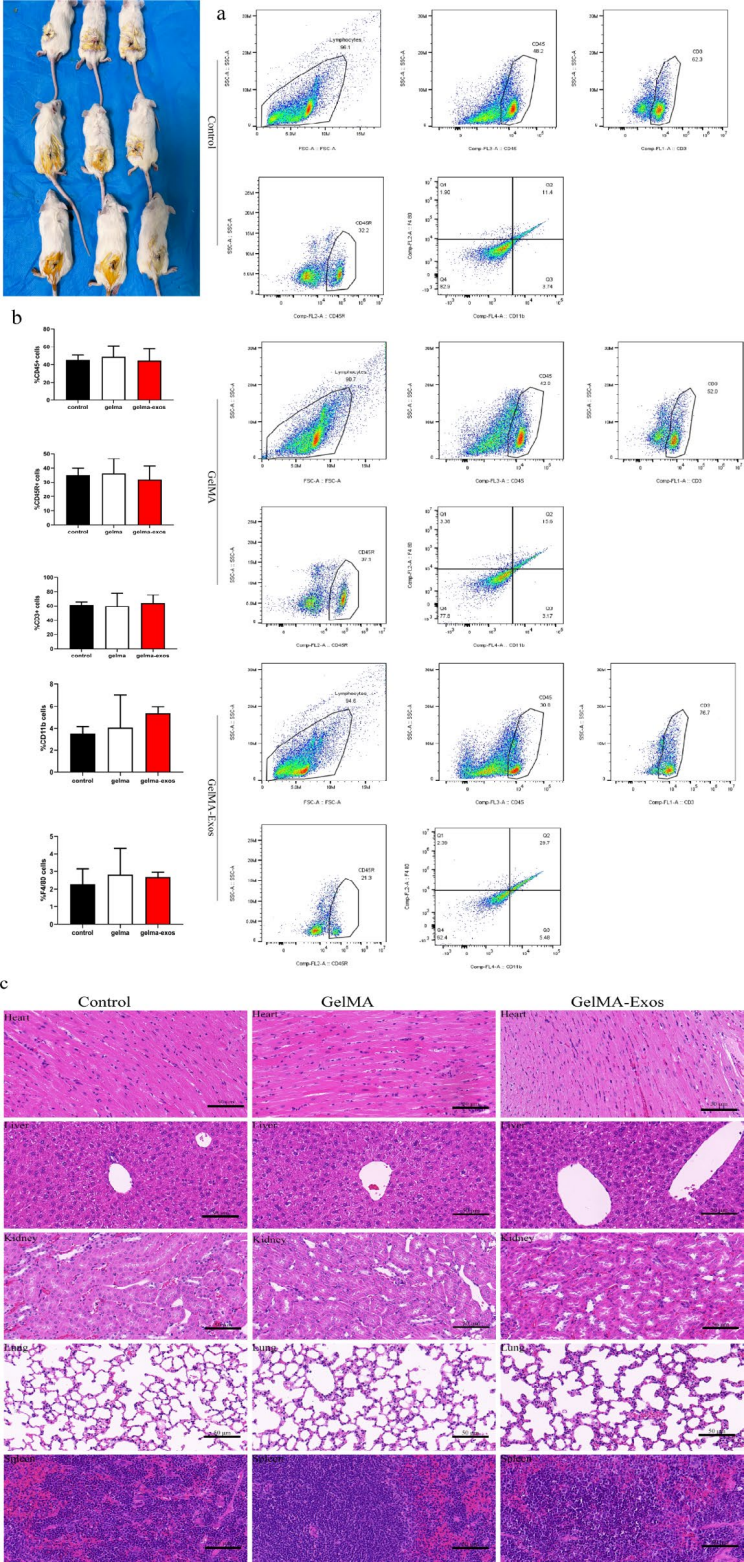
Biocompatibility of methacrylated gelatin (GelMA) and GelMA-Exos exosomes. GelMA and GelMA-Exos were embedded separately under the skin of mice. Seven days later, spleens and other major organs of the mice were collected, and immune cells were tested. **a** Flow cytometric analysis of immune cells in mouse spleens seven days after the subcutaneous implantation of gels. **b** Quantitative analysis of immune cells in the mouse spleen. **c** HE staining results of the major organs in mice

Flow cytometry was used to analyze the immune cells in the spleens of mice in each group (Fig. 4a). No statistically significant difference was observed between the white blood cells, T lymphocytes, and macrophages in the spleen of mice in the GelMA-Exos group and those in the control group, suggesting that GelMA-Exos did not trigger immune rejection (Fig. 4b). Hematoxylin and eosin (HE) staining results of different groups of heart tissues showed that the myocardial fibers were neatly arranged, the nucleus was clear and complete, and there were no obvious symptoms of apoptosis (Fig. 4c). HE staining results of different groups of liver cells indicated that the cells were arranged closely and in order, the bile duct was clearly distributed, and no obvious fibrosis and necrosis were observed (Fig. 4c). There was no significant inflammatory cell infiltration in the spleen of different groups after HE staining (Fig. 4c). HE staining results of different groups of lungs showed that the alveoli in the lung tissue were round or oval, and the alveolar epithelial cells were regular and closely arranged (Fig. 4c). HE staining showed that the renal structure as well as the glomerular size and shape were normal and the boundary was clear (Fig. 4c). In summary, a comparison of the HE staining results of mice subcutaneously implanted with GelMA and GelMA-Exos in various organs demonstrated their non-toxicity in mice.

### Release of GelMA-Exos and their uptake by human umbilical vein endothelial cells (HUVECs)

**Fig. 5:**
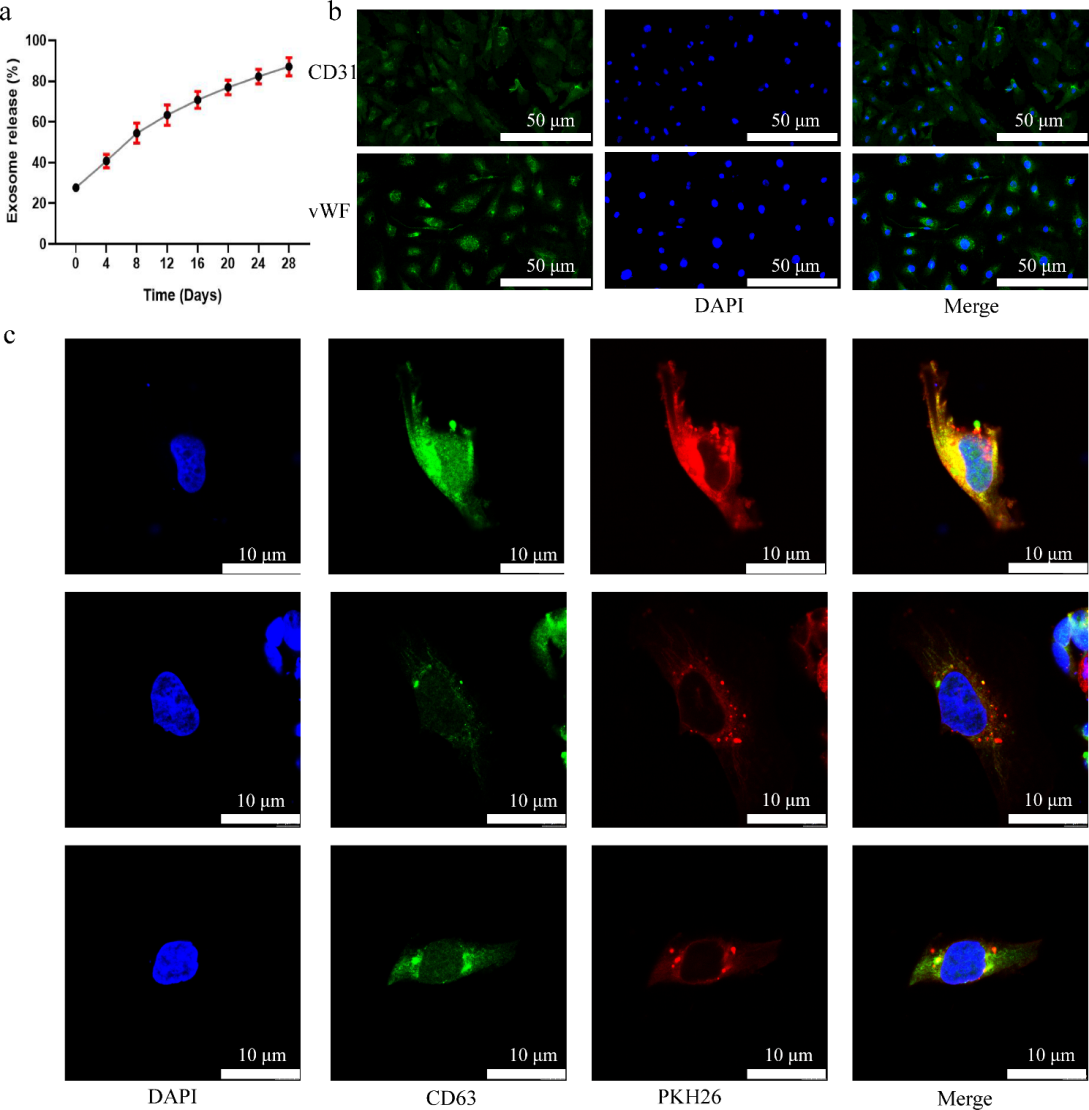
Controlled-release properties of exosomes from hydrogels and their uptake by HUVECs. **a** The BCA method was used to measure the controlled release curve of exosomes in GelMA-Exos. **b** Characteristic surface antibodies of HUVEC: CD31 and von Willebrand factor (vWF). **c** Immunofluorescence was used to observe the uptake of exosomes contained in GelMA-Exos by HUVECs. The nucleus was stained blue with DAPI, and the exosomes were stained red with PKH26. CD31, vWF, and CD63 was dyed green

We also determined the controlled-release capacity of GelMA-Exos (Fig. 5a). The release capacity of the hydrogels was determined by detecting the exosomes that had been released into the medium using a bicinchoninic acid (BCA) protein assay kit. We found that GelMA-Exos were released from hydrogels in vitro continuously for approximately 18–21 d. Then, GelMA-Exos were co-cultured with HUVECs. HUVEC is a pebble-like cell whose characteristic surface antibodies include CD31 and von Willebrand factor (vWF) (Fig. 5b). After incubation for 12 h, red fluorescent Paul Karl Horan (PKH)26 and green fluorescent CD63 double-dyed exosomes crossed the cell membrane into the cytoplasm of HUVECs and accumulated around the nucleus (Fig. 5c).

### GelMA-Exos promote the migration and proliferation of HUVECs

**Fig. 6:**
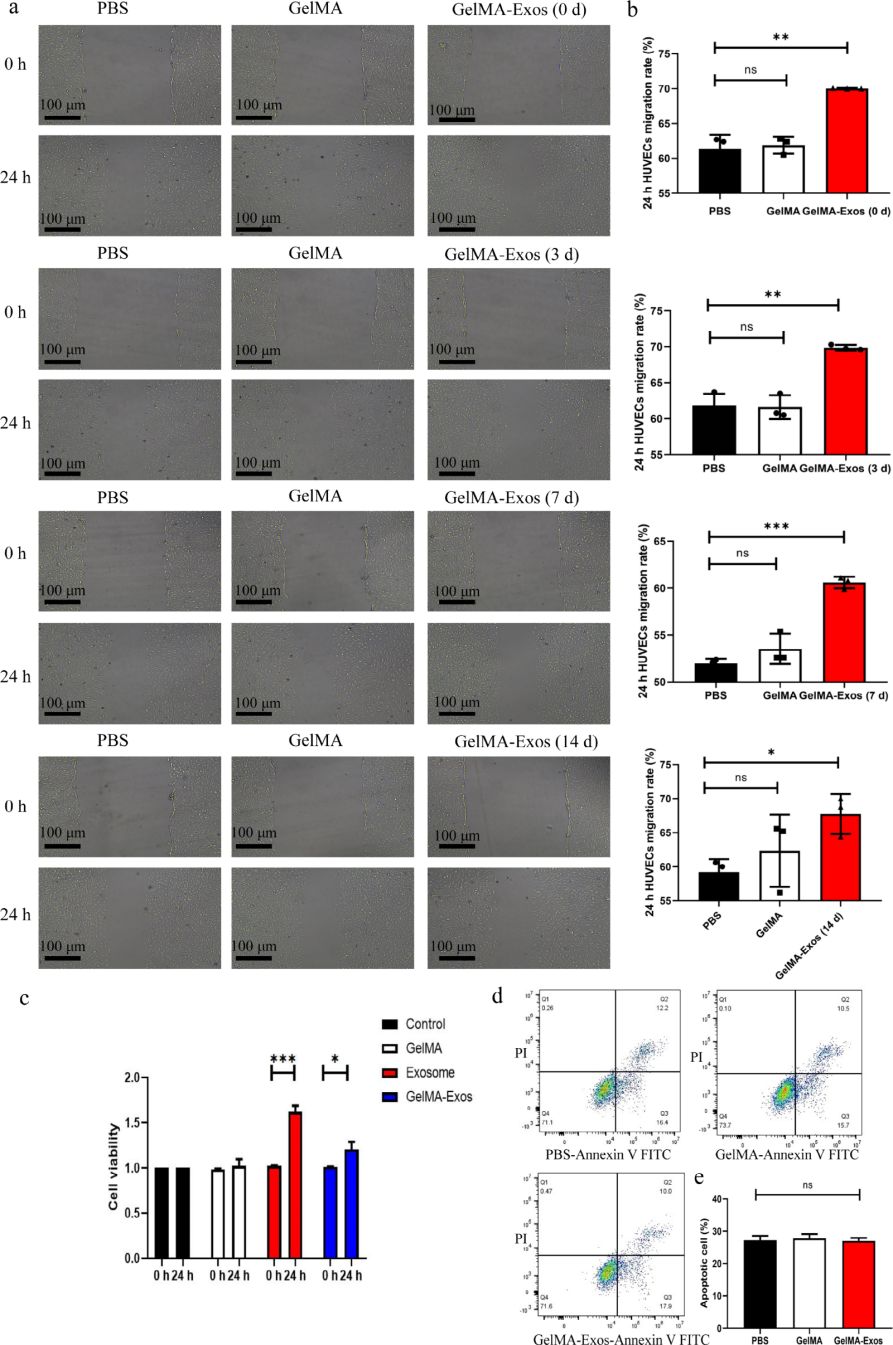
Methacrylated gelatin exosomes (GelMA-Exos) were found to promote the proliferation and migration of HUVECs. **a** Representative images of scratch wound assay performed at various time points after GelMA-Exos was soaked in PBS. **b** Quantitative analysis of the HUVEC migration area in each group at different time points. **c** CCK-8 assays of HUVEC proliferation. **d** Annexin V was used to detect the apoptosis of HUVEC cells. **e** Quantitative analysis of apoptotic proportion of HUVEC in each group. Data are presented as the mean ± SD. *: compared with control group, *P* < 0.05. ***: compared with control group, *P* < 0.01. HUVECs, human umbilical vein endothelial cells

To assess HUVEC migration, a cell scratch assay was performed. As indicated in Fig. 6a and b, the GelMA-Exos groups showed significantly increased cell migration abilities compared to those in the GelMA hydrogel and PBS groups. To further explore the advantages of GelMA-Exos, we also performed a migration assay for more periods. The results showed that GelMA-Exos still had the ability to promote HUVEC migration after 21 d of immersion in PBS (Fig. 6b). The results of the Cell Counting Kit-8 (CCK-8) assay also suggested that GelMA-Exos facilitated HUVEC proliferation, which was superior to that in the control and GelMA groups, indicating that the exosomes in the GelMA hydrogel could be partially released at an early stage and prolong their survival in vitro (Fig. 6c). Moreover, we analyzed the effect of GelMA-Exos on HUVECs by performing annexin V flow cytometry (Fig. 6d), and the results indicated that GelMA-Exos and the GelMA hydrogel did not increase HUVEC apoptosis (Fig. 6e).

### GelMA-Exos inhibit intimal hyperplasia in venous grafts

**Fig. 7:**
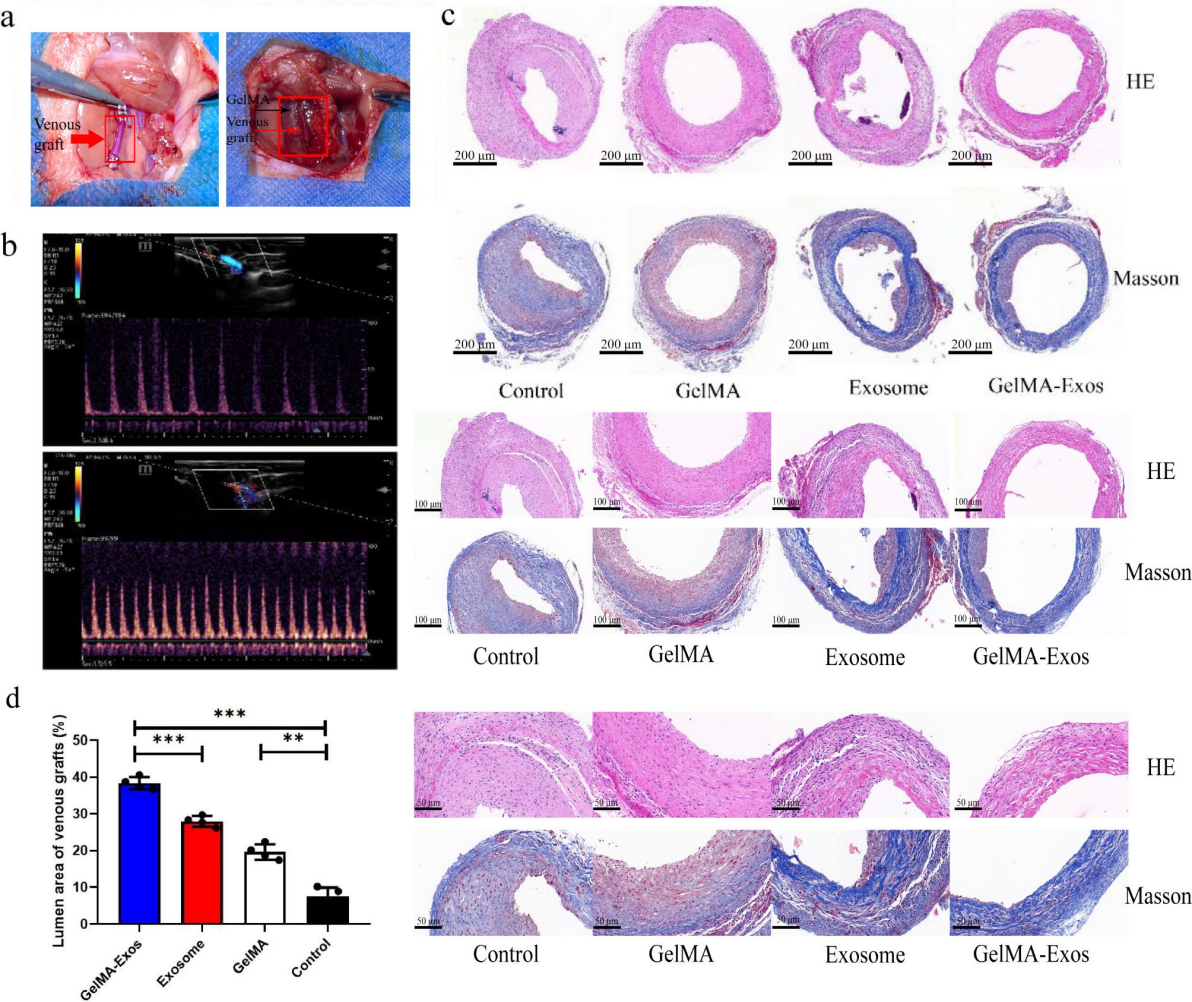
Establishment of venous graft model in rats and analysis of lumen area of venous grafts. **a** The rat vein graft model was constructed using the cannula method, and the methacrylated gelatin (GelMA) hydrogel was applied around the graft. **b** Color Doppler ultrasound was performed to detect the blood vessels of the venous graft. **c** HE and Masson staining of rat venous graft vessels. **d** Quantitative analysis of the lumen area of venous grafts in rats after 28 d

Our study constructed the jugular vein transplantation model and wrapped GelMA-Exos and GelMA around the vein graft (Fig. 7a). hUCMSC-Exos were injected into rats through the tail vein. Echocardiography was performed to examine the patency and effectiveness of the vein graft (Fig. 7b). Venous grafts were collected approximately 30 d after the transplantation procedure, and the tube wall of the grafts became thicker, while the lumen became narrower. Masson and HE staining revealed that the intima and media of the vein grafts thickened at 28 d after surgery, and the smooth muscle cells in the media migrated and proliferated excessively, resulting in narrowing of the lumen of the vein grafts. To compare the degree of stenosis, we calculated the ratio of the lumen area to the total vessel area. HE and Masson staining showed that the degree of vascular stenosis was significantly reduced in the exosome group compared with that in the control group (*P* < 0.05, Fig. 7c). The proportion of the vascular lumen area in the GelMA-Exos group was also significantly increased compared with that in the control (*P* < 0.05, Fig. 7d) and GelMA (*P* < 0.05, Fig. 7c) groups.

### Detection of the endothelial process using immunofluorescence

**Fig. 8:**
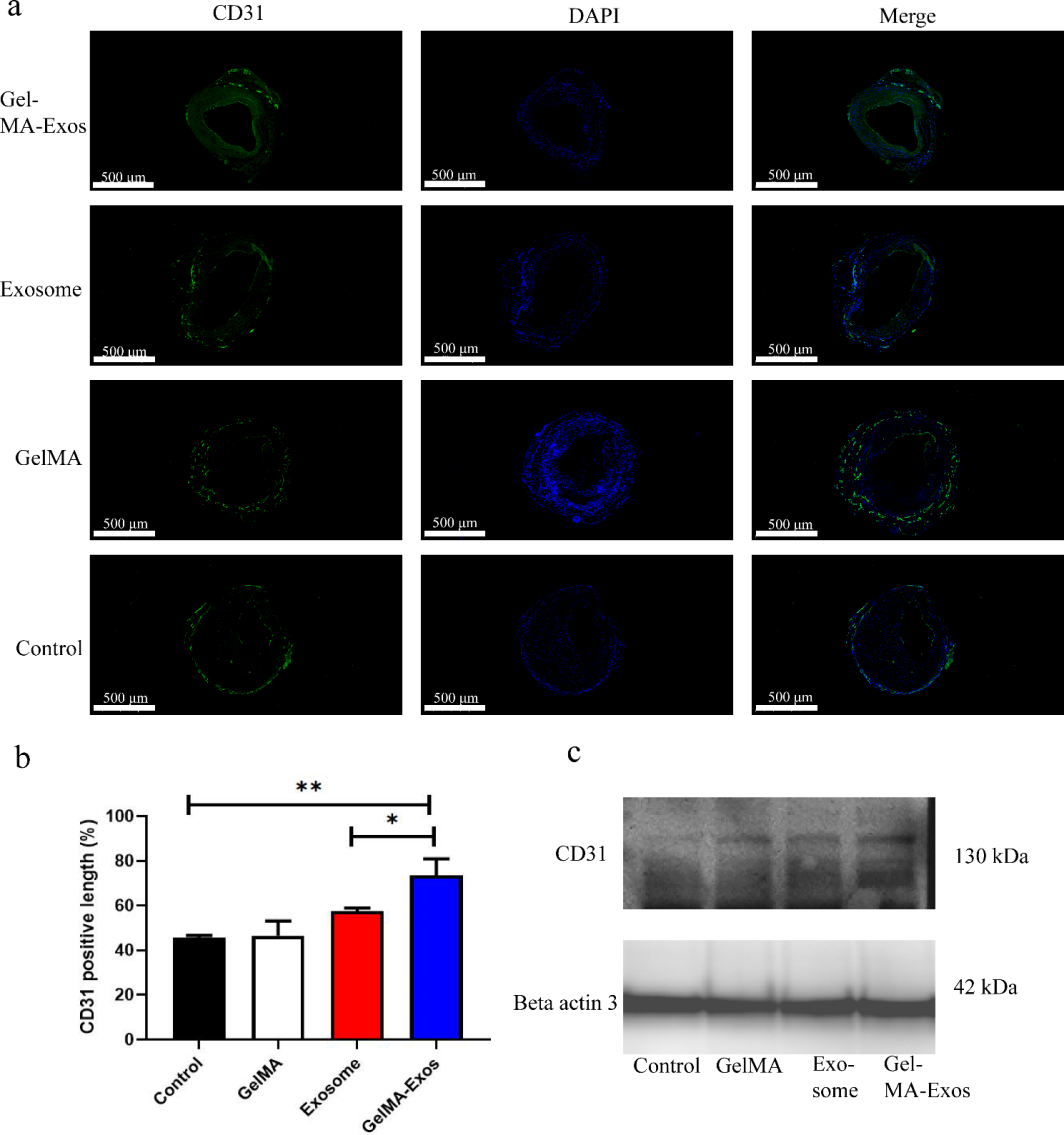
Analysis of degree and course of venous graft reendothelialization via immunofluorescence. **a** CD31-positive immunofluorescence distribution on the luminal surfaces of venous grafts, green. **b** Quantitative analysis of the coverage length ratio of CD31-positive cells in each group. **c** Western blot analysis of total CD31 in venous grafts

**Fig. 9:**
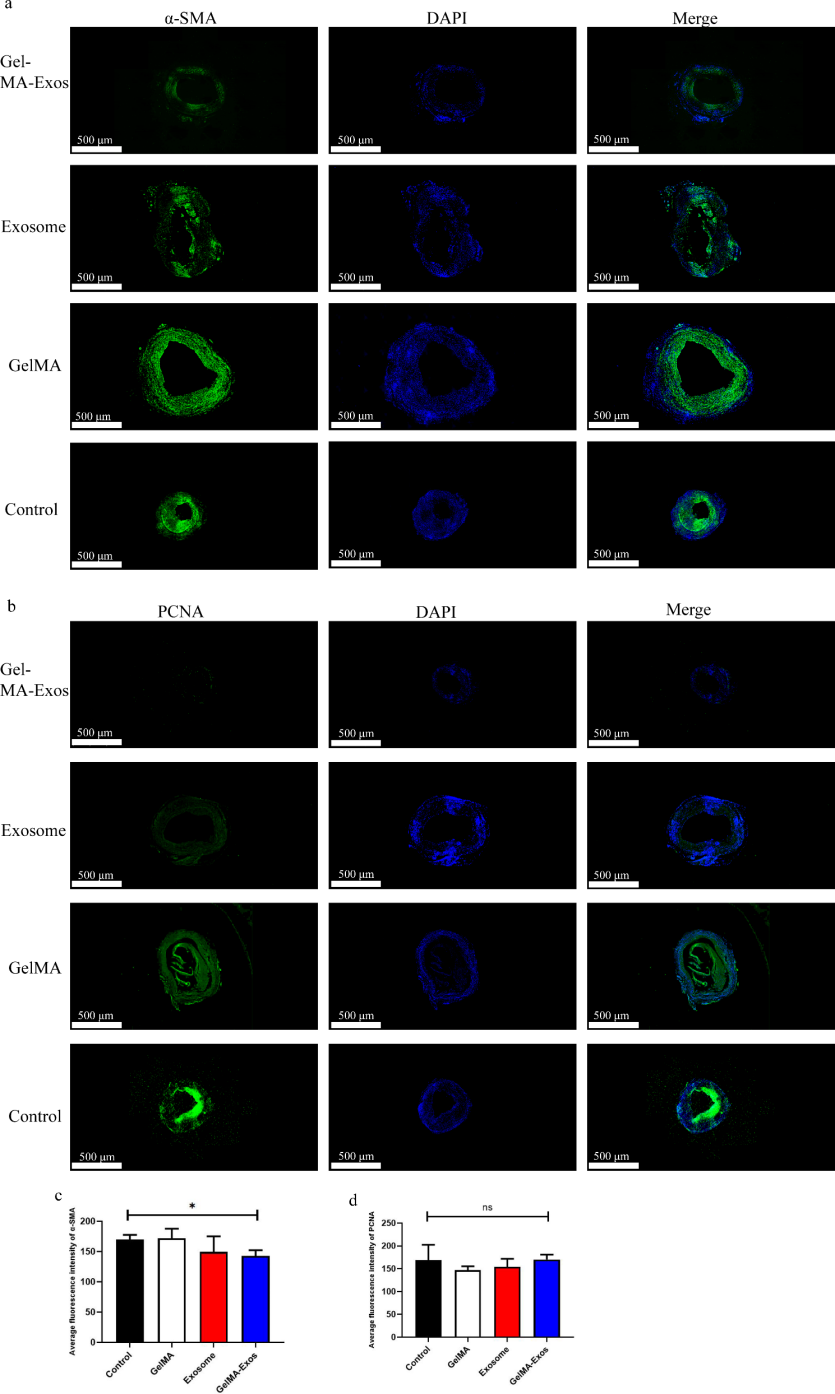
Analysis of the proliferation of smooth muscle cells via immunofluorescence. **a** Immunofluorescence distribution of α-SMA-positive cells in venous grafts (green). **b** Immunofluorescence distribution of PCNA-positive cells in venous grafts (green). **c** Quantitative analysis of the average fluorescence intensity of α-SMA in each group. **d** Quantitative analysis of the average fluorescence intensity of PCNA in each group

To investigate whether hUCMSC-Exos and GelMA-Exos could promote endothelial growth, we performed immunofluorescence analysis of endothelial cell surface markers CD31 at 28 d after vein graft. The GelMA-Exos and exosome groups showed positive immunofluorescence staining for CD31 along the lumen of the venous graft (Fig. 8a). Immunofluorescence showed that the length of the lumen wall in the GelMA-Exos group was mostly covered by CD31-positive cells, indicating that the degree of reendothelialization is the highest in this group (Fig. 8b). Moreover, western blot analysis of CD31-positive cells from different groups of venous grafts suggested that CD31 expression levels in the GelMA-Exos group were higher than those in the control group (Fig. 8c). Simultaneously, we also found that the reendothelialization effect of GelMA-Exos was the best among the groups. Furthermore, we investigated the expression of smooth muscle alpha-actin (α-SMA) (Fig. 9a) and proliferating cell nuclear antigen (PCNA) (Fig. 9b) in each group of venous grafts via immunofluorescence. The results indicated that the fluorescence intensities of α-SMA (Fig. 9c) in the GelMA-Exos group were lower than those in the control group.

### Key regulatory pathways associated with the reendothelialization of venous grafts

**Fig. 10:**
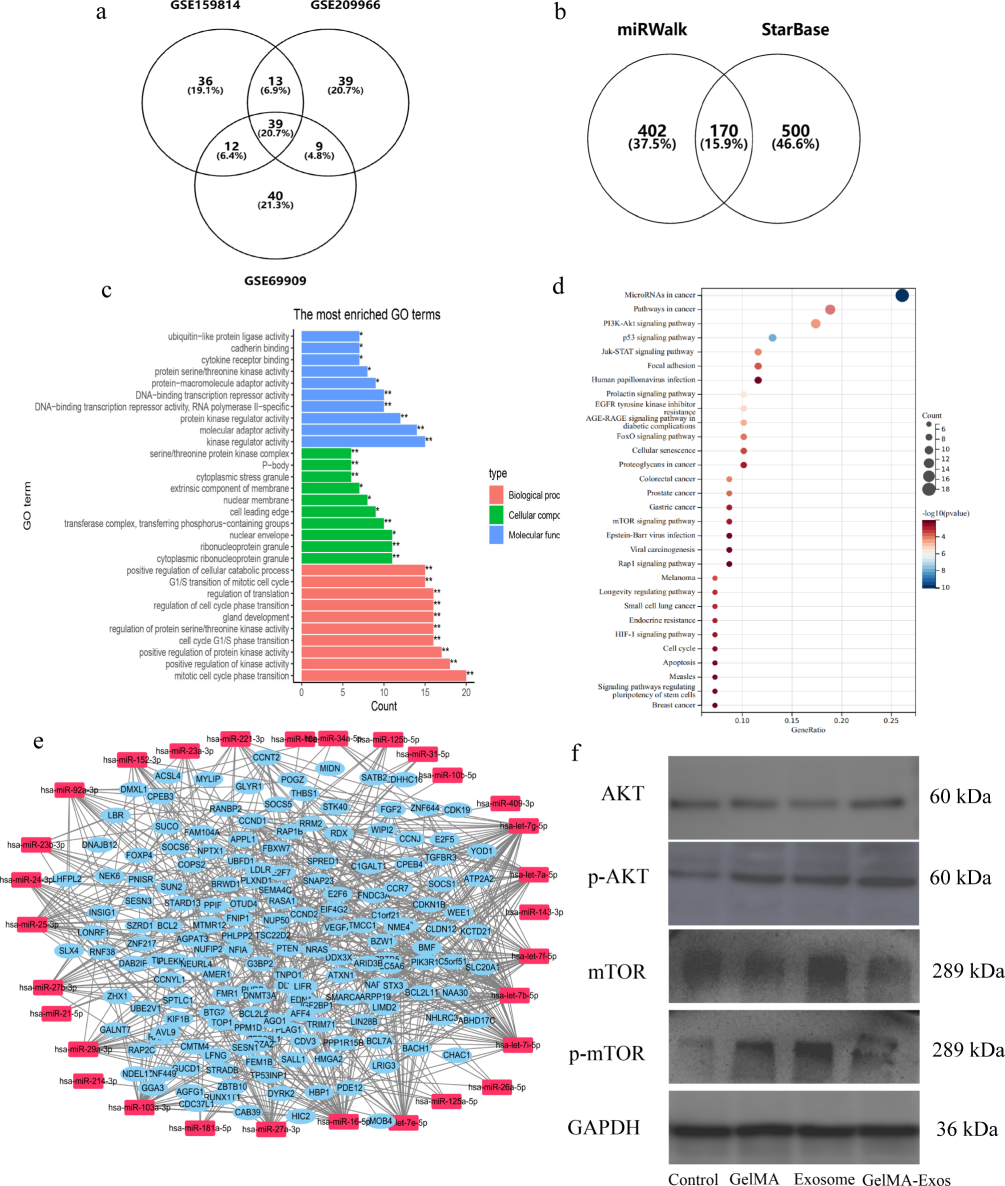
Bioinformatics analysis of the mechanism through which hUCMSC exosomes regulate HUVECs. **a** Top 100 miRNAs from three different datasets in the database, with the intersection taken. **b** Two algorithms, miRWalk2 and Starbase2, were used to predict mRNA, and the intersection was taken. **c** Visual results of GO analysis. **d** Bubble map of KEGG signaling pathways (gene count ≥ 5). **e** Interactions among 170 genes predicted based on the STRING database, with results visualized. **f** Western blot analysis of the involved signaling pathways. hUCMSCs, human umbilical cord mesenchymal stem cells. HUVECs, human umbilical vein endothelial cells

For each dataset, we converted the miRNA expression data from counts to transcript per million (TPM) values, averaged the miRNA expression in the sample, sorted the mean value from largest to smallest, and selected miRNAs with expression levels in the top 100. To make the results more reliable, we considered the intersection of the top 100 miRNAs of the three datasets, resulting in a total of 39 miRNAs (Fig. 10a). These 39 miRNAs were considered to have a leading role in the effects of hUCMSC-Exos and were used for subsequent studies.

In this study, two algorithms, miRWalk2 and Starbase2, were used to predict messenger RNA (mRNA)–miRNA interactions. For miRWalk2, we screened the miRNA–mRNA interactions predicted using both TargetScan and miRDB. For Starbase2, we screened for miRNA–mRNA interactions using the following parameters: clipExpNum ≥ 10, predicted sum > 5, and pancancerNum > 5. We compared the mRNA predicted by the two algorithms and obtained 170 mRNAs (Fig. 10b).

Gene Ontology (GO) function and Kyoto Encyclopedia of Genes and Genomes (KEGG) pathway enrichment analyses of 170 mRNAs were performed. For the GO analysis, we selected the top 10 terms for the presentation (Fig. 10c). For the KEGG analysis, we selected channels with a gene count ≥ 5 to display the bubble map (Fig. 10d). Finally, we used the Search Tool for the Retrieval of Interacting Genes/Proteins (STRING) database to predict the interactions among the 170 genes, and Cytoscape (3.6.1)was used to visualize miRNA–mRNA and mRNA–mRNA interactions (Fig. 10e). In this study, we found that signaling pathways related to cell migration and proliferation, including p53, phosphoinositide 3-kinase (PI3K)–AKT, and mammalian target of rapamycin (mTOR), might play important roles in the effects on endothelial cells. Furthermore, we found evidence of increased p-mTOR and p-AKT protein expression in the rat vein transplantation model (Fig. 10f). According to Western-blot calculation by ImageJ software, we found that the expression levels of AKT and mTOR proteins in each group were similar, suggesting that the AKT-mTOR signaling pathway may be involved in the regulation of endothelial cell function (Fig. 10f).

## Discussion

Venous graft restenosis after coronary artery bypass transplantation is mostly generated by intimal hyperplasia (IH), which is a key problem that needs to be addressed (21). After heart bypass surgery, the venous grafts are subject to complex physiological changes. First, the structure and function of endothelial cells are impaired, inflammatory factors are released, and white blood cells and platelets accumulate in this area (1). Subsequently, vascular smooth muscle cells proliferate and migrate to the graft vessels to adapt to the increased vascular shear stress. This process is accompanied by a sharp decrease in the expression levels of various vasodilator factors and nitric oxide (22). Without the protection of endothelial cells, the smooth muscle, directly exposed to the blood, will proliferate excessively under the stimulation of inflammatory factors, resulting in stenosis of the graft lumen (23). Therefore, repairing and maintaining the function of vascular endothelial cells acts as a pivotal role in inhibiting IH in the early and middle stages of vein transplantation.

MSCs are a prospective treatment option for tissue repair and regeneration in various cardiovascular diseases (24). In addition, there is increasing evidence that stem cell therapy is effective through signal transduction pathways mediated by EVs (25). Exosomes contain a variety of small molecules that carry biological information, such as miRNA and proteins, which are the core substances mediating intercellular communication and can even regulate cells of different species (26). Currently, the therapeutic efficacy of stem cell-derived exosomes has been demonstrated in various animal disease models (27). However, the rapid clearance and easy destruction of exosomes limit their application in some disease models. It is possible to promote the application of exosomes in disease models based on the development and utilization of various biological materials (28). Several studies have applied injectable hydrogels and exosomes to various disease models, such as intervertebral disc degeneration and cartilage regeneration, because hydrogels can effectively protect and prolong the half-life and function of exosomes in the body (29).

In this study, GelMA was combined with hUCMSC exosomes and applied to a rat vein graft model to inhibit graft restenosis. Moreover, our study also demonstrated that hUCMSC-Exos might stimulate the proliferation and migration of vascular endothelial cells by activating mTOR and PI3K–AKT signaling pathways. After the completion of gel synthesis, the surface of GelMA was observed using SEM and was found to be smooth. GelMA also preserved and prolonged the release of exosomes. Moreover, GelMA-Exos did not stimulate the spleen with an increase in immune cells in mice, demonstrating that GelMA-Exos did not elicit an immune response. The biological safety of GelMA-Exos was further confirmed by the HE staining results of the main internal organs in mice. Masson staining of the blood vessels in the graft specimens showed that the stenosis was eccentric and uneven. Furthermore, we confirmed the increased expression of α-SMA in vein grafts via immunofluorescence. Notably, we also observed increased expression of the endothelial cell-specific antigen CD31 in the GelMA-Exos and Exo groups using an immunofluorescence assay. The results of these experiments all proved that wrapping GelMA-Exos around the venous graft achieved the best effect of inhibiting restenosis.

Endothelial cell layer can maintain the integrity of the vessel wall by providing a barrier function, limiting SMC proliferation and migration, and promoting blood vessels to protect the medium, such as through nitric oxide synthesis, which is very important (30). Therefore, the enhanced reendothelialization of the damaged vein can effectively prevent lumen stenosis of the vein graft, and the process of reendothelialization mainly depends on the migration and proliferation of vascular endothelial cells (31). In vitro cell experiments and membrane dye labeling demonstrated that hUCMSC-Exos could be released from GelMA-Exos and be taken up by HUVECs. This is compatible with the results of Liang’s studies, suggesting that exosomes secreted by fat-derived MSCs are taken up by HUVECs (3). Previous studies have shown that hUCMSC-Exos mediate the expression of vascular endothelial growth factors in endothelial cells through mitogen‑activated protein kinase (MAPK)–extracellular signal-regulated kinase 1/2 (ERK1/2) signaling pathways, thereby promoting reendothelialization (19). To further study the regulatory mechanism underlying the effects of hUCMSC-Exos on endothelial cells, we downloaded three datasets from the Gene Expression Omnibus (GEO) database, screened miRNAs that might play a major role, and analyzed and verified the possible regulatory mechanisms, including the mTOR and PI3K–AKT signaling pathways. Other studies have also reported that the PI3K–AKT–mTOR pathway is vital for cell proliferation and apoptosis (32). Pei et al. pointed out that activation of the PI3K–AKT–mTOR signaling pathway can promote angiogenesis and endothelial cell functions (33). Further, Liang et al. indicated that human bone marrow MSC-derived exosomes target angiogenesis and promote bone regeneration via the AKT/mTOR pathway (34). In our study, we detected increased expression of phospho- (p)-AKT and p-mTOR in the experimental group, suggesting that the PI3–AKT–mTOR signaling pathway can be activated by hUCMSC-derived exosomes.

We also unexpectedly found that the GelMA hydrogel, applied alone around venous grafts, could inhibit the intimal hyperplasia of blood vessels. This could be related to the mechanical properties and external support provided by the GelMA hydrogel. Hydrogels with mechanical properties can act as “extravascular scaffolds” for venous grafts, effectively limiting the overdilation of vascular bridges and hemodynamic changes (35). External vascular stents not only effectively prevent venous graft expansion but also regulate the shear stress of venous blood vessel walls, thus inhibiting intimal hyperplasia (36). Moreover, by limiting graft overdistension, the diameter of the venous graft can match the artery, thus keeping the blood flow rate stable and reducing the blood turbulence (37). This can significantly improve the intimal hyperplasia caused by hemodynamic disorders. Another benefit of extravascular stents is the promotion of angiogenesis around transplanted veins. When macroporous and loose-fitting stents are used, several neovascularization events can be observed around the transplanted vein (38). Currently, only a few extravascular stents can be applied in clinical practice, whereas the application of most extravascular stents is still in its infancy. Considering their convenience and practicality for clinical applications, extravascular scaffolds made of light-cured hydrogels are a promising therapeutic option. Hydrogel scaffolds loaded with traditional Chinese herbs, such as *Bletilla* and *Astragalus membranaceus*, have also been reported for the treatment of the restenosis of grafts, and good progress has been made (39).

Our study has certain limitations. First, the rat model of venous transplantation cannot fully simulate the complex physiological conditions that could occur after coronary artery bypass transplantation. Second, we need to elucidate the specific mechanisms and pathways involved in the regulation of vascular endothelial cell proliferation and migration mediated by hUCMSC exosomes or other intervening exosomes. Third, the exosomes used in this paper are of insufficient purity, and there may be a small number of microvesicles larger than 150 nm in diameter. Finally, extensive clinical trials are required to evaluate the biosafety of GelMA-Exos.

## Conclusion

In summary, we constructed a complex comprising hUCMSC-Exos and a GelMA photosensitive hydrogel that can be used to inhibit restenosis in venous grafts and extend their service life. GelMA can carry exosomes and control their slow release, promoting endothelial cell proliferation in the intima of venous grafts, thereby causing “reendothelialization” to prevent early intima proliferation. Bioinformatics analysis and western blotting showed that hUCMSCs-Exos may regulate the proliferation and migration of endothelial cells by activating the PI3-AKT-mTOR pathway. The application of GelMA-Exos composites did not cause immune rejection or toxicity in various organs (heart, liver, lung, and kidney) in the mouse models. Therefore, this biomaterial-based exosome therapy is a promising treatment for vein graft restenosis.

## Materials and methods

### Animals

All animal experiments involved in this study met the requirements and standards of animal experimental ethics (protocol number: 2019-N(A)-086). Sprague–Dawley (SD) rats (age: 12–13 weeks; weight: 150–230 g; n = 86) were purchased from the Animal Center, Anhui Medical University. Before the experiment, 90 female rats were acclimated at 20–25 ℃ for one week with a relative humidity of 35–65%. Female and male mice were purchased from the Animal Experimental Center of Anhui Medical University. Each mouse was aged around six to eight weeks and weighed 30–40 g.

### hUCMSC and HUVEC culture

hUCMSCs were cultured in an incubator (HERAcell150i, Thermo, China) containing 5% carbon dioxide using α-Minimum Essential Medium (MEM) (Hyclone, USA) and identified via flow cytometry (CytoFLEX, Beckman, USA). In the flow identification process, the fluorescein antibodies involved included anti-CD73 (344,015, Biolegend, China), anti-CD105 (323,205, Biolegend, China), anti-CD90 (328,108, Biolegend, China), anti-CD14 (367,115, Biolegend, China), anti-CD19 (363,007, Biolegend, China), anti-HLA-DR (307,605, Biolegend, China), anti-CD34 (343,505, Biolegend, China), and anti-CD45 (368,503, Biolegend, China). Adipose and chondrogenic differentiation media (Gibco, USA) were used to measure the differentiation ability of hUCMSCs. HUVECs were identified using immunofluorescence staining. The antibodies involved included anti-CD31 (342,553, Biolegend, China) and anti-vWF (371,979, Biolegend, China). HUVECs were cultured in a endothelial cell medium (ScienCell, USA) supplemented with 5% fetal bovine serum.

### Exosome extraction and identification

Exosomes were obtained via rapid centrifugation. First, the cell culture supernatants of hUCMSCs (cells passaged three – five times) were collected and centrifuged at 2,000 × *g* for 10 min and dead cells were removed. For the remaining supernatant, the centrifugal force was increased to 10,000 × *g* for 30 min to remove cell debris, and the treated supernatant was then ready for centrifugation. Crude exosome precipitates were obtained via centrifugation at 100,000 × *g* for 75 min. Exosomes were precipitated with PBS again at 100,000 × *g* and centrifuged for 75 min, and purified exosomes were obtained. Finally, the extracted exosomes were resuspended using 200 μL of cold PBS. The morphology of hUCMSC-Exos was observed via TEM (JEM-ARM300F, JEOL, Japan). The size of hUCMSC-Exos and expression of surface markers (CD63, CD9, and CD81) were determined using a NanoSight (Malvern, England) and through western blotting, respectively.

### Synthesis of GelMA-Exos hydrogel

Based on previous literature reports, we synthesized GelMA (12). To 50 mL of PBS, 10 g of gelatin and 8 mL of methacrylate were added individually and thoroughly mixed. The solution was diluted to terminate the chemical reaction of gelatin with methacrylate and then dialyzed with double-distilled water (DD-H_2_O) for five days. The solution was then freeze-dried and stored at − 20 °C.

The GelMA-Exos hydrogel was prepared as follows: First, a solution of the photoinitiator lithium phenyl-2,4,6-trimethylbenzoylphosphinate (LAP) was prepared, and GelMA was dissolved in LAP solution at a 25% (w/v) concentration and heated in a water bath at 40 ℃ for 20 min. Next, the exosome solution (300 μg of exosome protein suspended in 200 μL of PBS) was added and stirred for 30 s. GelMA-Exos solution was irradiated under ultraviolet light (wavelength 405 nm) for 40 s and stored at − 20 °C after gelation.

### Hydrogel degradation assay

GelMA and GelMA-Exos were added to 5 mL of PBS and incubated at 37 °C. At five days intervals, the gel was removed from the PBS, freeze-dried, and weighed using a microbalance (Constant, China). Female and male mice were purchased from the Animal Experimental Center of Anhui Medical University. Each mouse was aged around six – eight weeks and weighed 30–40 g. After anesthetizing the mice, the skin was sterilized and cut from the backs of the animals. The GelMA hydrogel and GelMA-Exos were sterilized and implanted into the skin tissue on the backs of mice. The skin incision was then re-sutured. After 28 d, GelMA and GelMA-Exos were removed and weighed.

### Biocompatibility experiments with GelMA-Exos

After domesticating the mice as described previously herein, an incision was made in the backs of the animals. The 18 mice were randomly divided into three groups: control group, GelMA group, and GelMA-Exos group. There were six mice in each group. GelMA-Exos and GelMA of similar volumes and weights were placed under the skin, whereas in the control group, the skin was only cut and then re-sutured. After 28 d, the mice were euthanized, and their hearts, lungs, livers, spleens, and kidneys were removed for subsequent experiments. HE staining was performed on the hearts, lungs, livers, spleens, and kidneys of mice (n = 3). Three sections of different parts of each mouse were taken from different organs, and a total of 45 tissue sections were analyzed. Then, we used a microscope (X200) (Nikon TS2, Nikon, Japan) to observe the different tissue sections. The spleens of the mice were removed separately and rinsed with PBS. A single-cell suspension was prepared by placing mouse spleens on a 100-target cell screen; they were then subjected to pressing and grinding on the cell screen using a syringe needle core for 30 s, and the screen was washed by adding 10 mL of sterile PBS based on three batches. Immune cells in the single-cell suspension were collected via centrifugation (200 × *g*, 10 min). The cells in each group were precipitated with 0.5 mL of PBS, 3 μL of flow cytometry (CytoFLEX, Beckman, USA) antibodies were added, and the sample was incubated at 4 ℃ for 15 min in the dark. The antibodies used included anti-CD45 (45-0451-80, Invitrogen, USA), anti-CD3 (100,203, Biolegend, China), anti-CD45R (100,203, Biolegend, China), anti-CD11B (17-0118-41, Invitrogen, USA), and anti-F4/80 (12-4801-80, Invitrogen, USA). Flow cytometry (Beckman, Germany) was then performed.

### Characterization of GelMA-Exos and GelMA

To characterize the surface morphology of GelMA and GelMA-Exos, SEM images were obtained using a Sigma 300 field emission scanning electron microscope (EVO10, Zeiss, German). Hydrogel samples were prepared and freeze-dried overnight. Then, black double-sided tape was used to secure the hydrogel to the work surface. The surface of the sample was coated with gold through argon sputtering for a few seconds. Images were then captured. GelMA and GelMA-Exos were adequately dried using a freeze-dryer, and the dried products were collected and milled into a powder. Both GelMA and GelMA-Exos were characterized using Fourier-transform infrared spectrophotometry (Nicolet™ iS50, Thermo, USA). The rheological properties of the GelMA hydrogel, including the G’ and G’’, were determined using a rheometer (RheolabQC, Anton Paar, China). The GelMA hydrogel was scanned using an X-ray diffractometer (Smartlab SE, Rigaku, Japan) and then analyzed and mapped using ORIGIN 2019 software.

### Release kinetics of GelMA-encapsulated hUCMSCs‑Exos

To detect the release of hUCMSCs-Exos from GelMA in vitro, we mixed 100 mg of hUCMSCs-Exos with 20% GelMA at 4 °C and placed them in the upper compartment of a transwell, with 100 μL of PBS added to the lower wells, incubated at 37 °C. The PBS solution was removed from the lower compartment four days apart, and the BCA method was used to detect the protein concentration in PBS solution and calculate the levels of released exosomes.

### Uptake of hUCMSC-Exos

Exosomes were stained and labeled using the Paul Karl Horan (PKH)26 kit (Sigma, USA). Exosomes were fluorescently double-stained with an anti-CD63 (Sigma, USA) antibody. The 25% GelMA was mixed with double-labeled exosomes in a water bath. HUVECs were inoculated in 12-well plates at approximately 10^5^ cells per well. HUVECs were continued to co-culture with GelMA-Exos for 12 h. The nucleus of HUVECs was labeled using a 4′,6-diamidino-2-phenylindole (DAPI) (1:1,000) solution. Random imaging and the observation of HUVECs in 12-well plates were performed using a confocal microscope (Power HyD, Leica®, Germany).

### Surgical procedure

The animal vein graft model used in this study was constructed according to the literature (2). The surgical procedure used was as follows: SD rats were anesthetized intraperitoneally and heparinization was induced. The necks of SD rats were cut (approximately 1 cm) along the direction of the trachea, and unilateral veins were isolated. Next, the jugular vein was grafted onto the carotid artery. After venous transplantation, the rats were fed warfarin water according to their body weight. To study the combined effects of hUCMSC exosomes and GelMA, SD rats were divided into four groups as follows: vein graft, vein graft + GelMA (smeared around the graft), vein graft + exosomes, and vein graft + GelMA-Exos. Four weeks after transplantation, the surgically isolated venous grafts were retained, and the rats were euthanized.

### Color doppler ultrasound

After anesthesia, the hair removal process was carried out on the necks of rats, and then, conductive gel was daubed near the original incision. An ultrasonic sensor probe (L9-3U; RESONA9, China) was used to find and locate the graft vein and observe whether it was unblocked.

### HE staining

Slices were paraffin-embedded, dewaxed, hydrated, and baked in an oven at 62 ℃ for 60 min. They were then soaked in xylene (10,023,418, China) three times, for approximately 10 min each time. Then, they were soaked in 100% ethanol twice for one minute each time and then soaked in 95%, 90%, 85%, 80%, 70%, and 50% ethanol for one minute each. A drop of hematoxylin staining solution (BASO, China) was added, and the sample was rinsed with tap water for five minutes; the blue color returned after soaking the sample in tap water for 15–30 min. The slices were immersed in a 1% hydrochloric acid ethanol solution for approximately 5–30 s, rinsed again in tap water to restore the blue color, and then stained with 0.5% eosin ethanol solution (BASO, China) for one – three minutes. The slices were immersed in absolute ethanol three – five times and dried at 25 ℃. The slices were allowed to dry thoroughly, placed in xylene for five minutes, and observed after sealing.

### Masson staining

The vascular tissue samples were fixed with 4% paraformaldehyde, sliced after paraffin embedding, stained with Weigert iron hematoxylin for five minutes, rinsed with running water, differentiated in 1% alcohol hydrochloride for 10 s, and rinsed with tap water for five minutes to return to blue. The sample was stained with ponceau fuchsin stain solution for five minutes and washed with weak acid working solution for one minute. Phosphomolybdic acid aqueous solution was treated for three minutes. Aniline blue solution was re-dyed for five minutes. The sample was dehydrated and sealed for microscopic examination.

### Immunofluorescence staining

The samples were soaked in 4% paraformaldehyde solution and then soaked in 0.3% TritonX-100 (Sigma, USA) at room temperature for 15–20 min. The TritonX-100 was then removed and washed with PBS. Then, 0.25 g of bovine serum albumin (BSA) was added to 5 mL of PBS to prepare the sealing solution, and the sample was incubated in the sealing solution at 37 ℃ for 30 min. The primary antibody was added to the sealing solution and incubated at 4 ℃ overnight. The following primary antibodies were used: anti-α-SMA, anti-PCNA, and anti-CD31 (1:1,000, Beyotime, China). The primary antibodies on the remaining sections were washed with PBS, and then the secondary antibody (1:500, Beyotime, China) was added and incubated. The secondary antibodies were removed by washing once with PBS. A fluorescence quencher was added, and the cells were observed.

### Cell proliferation assay (CCK8)

HUVECs were collected by centrifugation, made into a single cell suspension, and the cell concentration was diluted to 5–10 × 10^4^ cells/mL. The cell suspension was gently mixed and added to the 96-well plate with 100 μL per well, and the edge holes were filled with sterile PBS. The inoculated 96-well plates were placed in an incubator and continued to grow until HUVECs covered the entire bottom of the wells. PBS, GelMA, exosomes, and GelMA-Exos were added to each well. After continued culture in the incubator for 24 h, 10 μL of CCK-8 (Bioss, China) was added to each well. HUVECs were continued to be cultured for four hours, and optical densities (OD) values of each well at 450 nm were measured using enzyme-labeler (Multiskan FC, Thermo, USA).

### Cell migration assay

GelMA-Exos was soaked in PBS and stored in a cell incubator. On day 0, day 3, day 7, and day 14 after soaking, the GelMA-Exos was removed and experimented. A marker was used to draw even horizontal lines approximately 0.5–1 cm apart on the back of the six-well plate. Approximately 5 × 10^5^ HUVECs were inoculated in each well and cultured overnight until cells completely covered the bottom of the plate. PBS, GelMA hydrogels, and GelMA-Exos were added separately to each well. With a ruler, cell scratches were made using a 20 μL pipette gun tip perpendicular to the well plate and line so that the scratch intersected the marked line. The head of the gun was vertical, to try to ensure that the width of each scratch was consistent. The cells were washed and added serum-free medium. Images were acquired after 24 h.

### Flow cytometric analysis of annexin V

HUVECs were collected and washed with cold PBS. Apoptosis was detected using Annexin V-fluorescein isothiocyanate (FITC)/propidium iodide kit (AP101, Multi Sciences, China). Dilution of 5× binding buffer to 1× binding buffer was performed with double steaming water. The cells were suspended with 500 μl 1× binding buffer. 5 μl FITC and 10 μl propyl iodide were added to each tube of cells. After gently mixing, the mixture was incubated for five minutes at room temperature. Analysis was performed using flow cytometry (CytoFLEX, Beckman, USA).

### Western blotting

The proteins were extracted with radioimmunoprecipitation assay (RIPA) and quantified using BCA. The proteins were separated and electrophoresed using 12% protein prefabricated glue (Beyotime, China), and then transferred to a Hybond-P polyvinylidene difluoride membrane. The membrane was removed after completion, put into 5% milk sealing solution, and sealed in a low-speed shaker at room temperature for one hour. After eluting the blocking solution, the primary antibody was added and incubated at 4 °C overnight. The antibodies used were anti-CD81 (1:1,000, Abcam, USA), anti-CD9 (1:1,000, Abcam, USA), anti-CD63 (1:1,000, Abcam, USA), anti-AKT (1:8,000, Abcam, USA), anti-p-AKT (1:8,000, Abcam, USA), anti-mTOR (1:5,000, Abcam, USA), and anti-p-mTOR (1:5,000, Abcam, USA). After eluting the primary antibody, the secondary antibody was added and incubated at 4 °C for 45 min. The strip position was analyzed after exposure.

### Bioinformatics analysis

Three datasets were downloaded from the GEO database as follows: GSE159814 (40), GSE209966 (41), and GSE69909 (42). The levels of miRNAs in the top 10–30%, in terms of expression, were screened from the three datasets, and the intersection was selected. This miRNA was considered the dominant miRNA in exosomes. Two algorithms, MiRWalk and TargetScan, were used to predict the interactions between miRNAs and mRNAs. The results of the two algorithms were combined to screen for genes that might be regulated by miRNAs. STRING (https://cn.string-db.org/) was used to analyze gene interactions, and a miRNA–mRNA–mRNA interaction network was established. Using the clusterProfiler package of R language (3.6), GO/KEGG analysis was used to analyze the functional enrichment of genes and explore pathways that could be involved in their regulation, with *P* < 0.05 considered statistically significant. We used the ggplot2 package to visualize the results.

### Statistical analysis

The data were expressed as the mean ± standard deviation and were processed using Statistical Product and Service Solutions (SPSS) version 21.0 software (Chicago, IL, USA). Fisher’s test of the minimum significant difference was used to compare two groups. *P* < 0.05 was considered statistically significant.

## Data Availability

All the data involved in this study are authentic and have been included in this published article.

## List of abbreviations

CABG: Coronary artery bypass grafting
EV: Extracellular vesicle
G’: Storage modulus
G’’: Loss modulus
GelMA: Methacrylated gelatin
HE: Hematoxylin and eosin
hUCMSC-Exos: Human umbilical cord mesenchymal stem cell-derived exosomes
IH: Intimal hyperplasia
miRNA: MicroRNA
MSCs: Mesenchymal stem cells
PBS: Phosphate buffered saline
SEM: Scanning electron microscopy
TEM: Transmission electron microscopy
